# Crystallization behavior of the Li_2_S–P_2_S_5_ glass electrolyte in the LiNi_1/3_Mn_1/3_Co_1/3_O_2_ positive electrode layer

**DOI:** 10.1038/s41598-018-24524-7

**Published:** 2018-04-18

**Authors:** Hirofumi Tsukasaki, Yota Mori, Misae Otoyama, So Yubuchi, Takamasa Asano, Yoshinori Tanaka, Takahisa Ohno, Shigeo Mori, Akitoshi Hayashi, Masahiro Tatsumisago

**Affiliations:** 1 Department of Materials Science, Graduate School of Engineering, Osaka Prefecture University, 1-1, Gakuen-cho, Naka-ku, Sakai, Osaka, 599-8531 Japan; 2 Global Research Center for Environment and Energy based on Nanomaterials Science, National Institute for Materials Science (NIMS), 1-1, Namiki, Tsukuba, Ibaraki, 305-0044 Japan; 3 Department of Applied Chemistry, Graduate School of Engineering, Osaka Prefecture University, 1-1, Gakuen-cho, Naka-ku, Sakai, Osaka, 599-8531 Japan

## Abstract

Sulfide-based all-solid-state lithium batteries are a next-generation power source composed of the inorganic solid electrolytes which are incombustible and have high ionic conductivity. Positive electrode composites comprising LiNi_1/3_Mn_1/3_Co_1/3_O_2_ (NMC) and 75Li_2_S·25P_2_S_5_ (LPS) glass electrolytes exhibit excellent charge–discharge cycle performance and are promising candidates for realizing all-solid-state batteries. The thermal stabilities of NMC–LPS composites have been investigated by transmission electron microscopy (TEM), which indicated that an exothermal reaction could be attributed to the crystallization of the LPS glass. To further understand the origin of the exothermic reaction, in this study, the precipitated crystalline phase of LPS glass in the NMC–LPS composite was examined. *In situ* TEM observations revealed that the *β*-Li_3_PS_4_ precipitated at approximately 200 °C, and then Li_4_P_2_S_6_ and Li_2_S precipitated at approximately 400 °C. Because the Li_4_P_2_S_6_ and Li_2_S crystalline phases do not precipitate in the single LPS glass, the interfacial contact between LPS and NMC has a significant influence on both the LPS crystallization behavior and the exothermal reaction in the NMC–LPS composites.

## Introduction

All-solid-state lithium batteries have recently attracted much attention owing to the use of nonflammable electrolytes^1^. In all-solid-state cells, the positive electrode layer is composed of positive electrode active materials and solid electrolytes. To improve the energy density and charge–discharge cycle performance, solid electrolytes exhibiting high ionic conductivity have been developed in recent years^2–7^. In particular, the sulfide-type Li_2_S–P_2_S_5_ glass electrolytes exhibit high conductivities of over 10^−4^ S cm^−1^ at room temperature. The characteristic features of the Li_2_S–P_2_S_5_ glass are that it crystallizes upon heat treatment, and its ionic conductivity strongly depends on the type of precipitated Li–P–S crystalline phase^8,9^. Typical examples of the superionic conducting crystalline phase are the Li_10_GeP_2_S_12_ analog (Li_3.25_P_0.95_S_4_) and Li_7_P_3_S_11_, both of which show ionic conductivities of over 10^−3^ S cm^−1^ at room temperature^10,11^. However, the crystallization of Li_2_S–P_2_S_5_ glasses involves an exothermic reaction, which affects the thermal stability of the all-solid-state cells when they are operated at a high temperature^12^. In a practical situation where the all-solid-state cells are utilized as a large power source for hybrid electric vehicles and accumulators, thermal stability is extremely critical.

All-solid-state batteries using Li_2_S–P_2_S_5_ glass electrolytes exhibit good energy density and cycle performance. In our previous study, we fabricated the all-solid-state cell In/75Li_2_S·25P_2_S_5_ glass (LPS)–LiNi_1/3_Mn_1/3_Co_1/3_O_2_ (NMC). The cell maintained good cycle performance as well as a solid interfacial contact between NMC and LPS during 20 charge–discharge cycles^13^. In terms of the thermal stability of the NMC–LPS composites, an exothermal reaction was present at approximately 200 °C, which was attributed to the crystallization of the LPS glass. In contrast, NMC did not directly contribute to the exothermic reaction because no structural change was detected during heating. The crystallization behavior of the LPS glass in the NMC–LPS composites was entirely different from that in the single LPS glass. The crystallization temperature of LPS glass significantly changed owing to the interfacial contact with the NMC. In addition, the glass transition of the LPS glass in the NMC–LPS composites was accompanied by the morphological shrinkage of the sample. These properties implied that the interfacial contact between NMC and LPS influence the LPS crystallization behavior and the corresponding exothermal reaction^13^.

In this study, we thus focused on the LPS glass in the NMC–LPS composites. To further understand the origin of the exothermic reaction present in the NMC–LPS composites, the precipitated crystalline phase in the LPS glass was examined using a computer program called “ProcessDiffraction.” In ProcessDiffraction, the electron diffraction (ED) patterns of polycrystalline and amorphous samples can be analyzed^14–17^. On the basis of the *in situ* TEM observations and first-principles calculation for the chemical reaction, the influence of the interfacial contact between NMC and LPS on the LPS crystallization behavior and origin of the exothermal reaction are discussed.

## Results and Discussion

**Figure 1:**
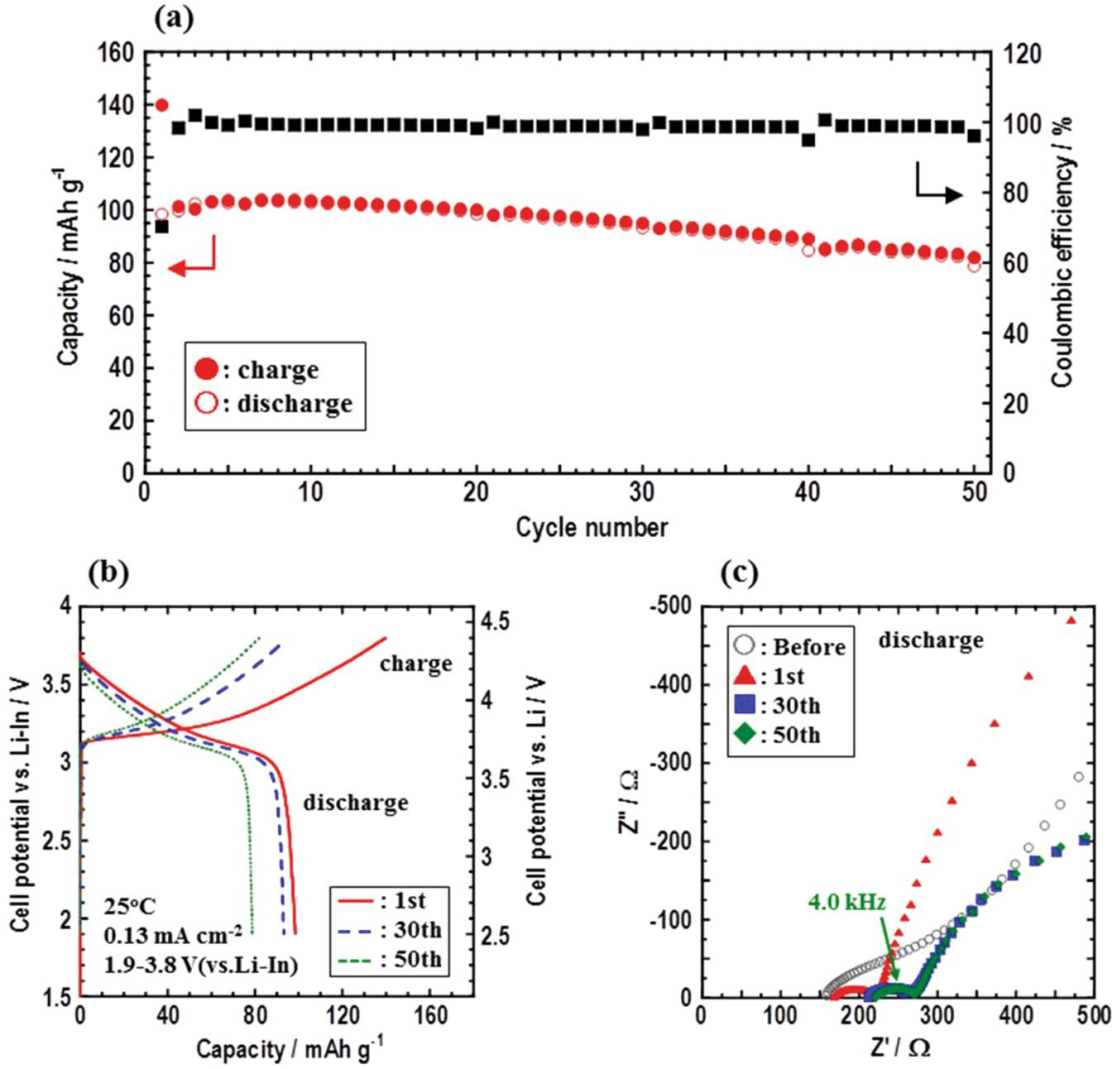
(**a**) Long-term cycle performances of the all-solid-state cell In/LPS/NMC, (**b**) Charge–discharge curves of the cell at 25 °C under a current density of 0.13 mA cm^−2^, and (**c**) Nyquist plots of the cell before charge–discharge and after the 1st, 30th, and 50th discharge cycles.

Firstly, electrochemical performances during long-term cycling of the fabricated all-solid-state cells In/LPS/NMC (NMC:SE = 75:25 in weight ratio) were investigated. Figure 1(b) shows the initial, 30th, and 50th charge–discharge curves of all-solid-state cells using the NMC-LPS composites at the current density of 0.13 mA cm^−2^. These cells are operated at a temperature of 25 °C in the voltage range of 1.9–3.8 V vs. Li–In. Figure 1(a) shows cycle performance of the cell. In spite of an irreversible capacity of approximately 40 mAh g^−1^ at the initial cycle, the cell maintains more than 80 mAh g^−1^ even after the 50th charge–discharge cycle. Under this charge–discharge condition, the fabricated cell using the NMC-LPS composites exhibits excellent stability during long-term cycling. Figure 1(c) shows Nyquist plots of the cell. A depressed semicircle (characteristic frequency of 4.0 kHz) is observed in the Nyquist plot after the initial charge-discharge measurement and the size of it is almost the same after the 50th discharge cycle.

**Figure 2:**
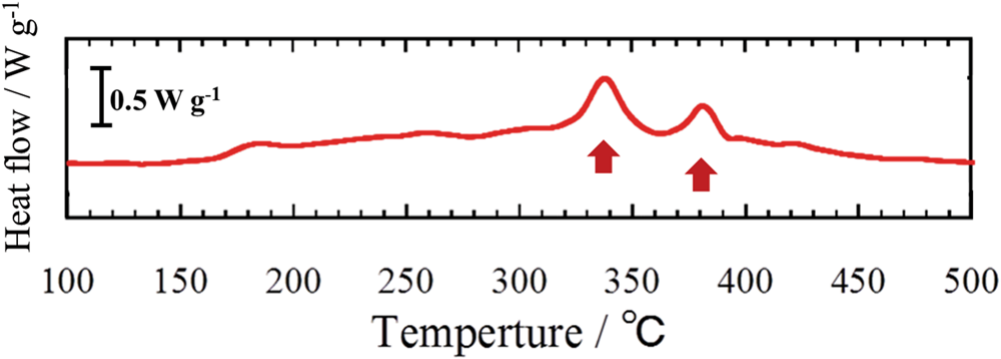
The DSC curve for the NMC–LPS composites after the initial charge.

**Figure 3:**
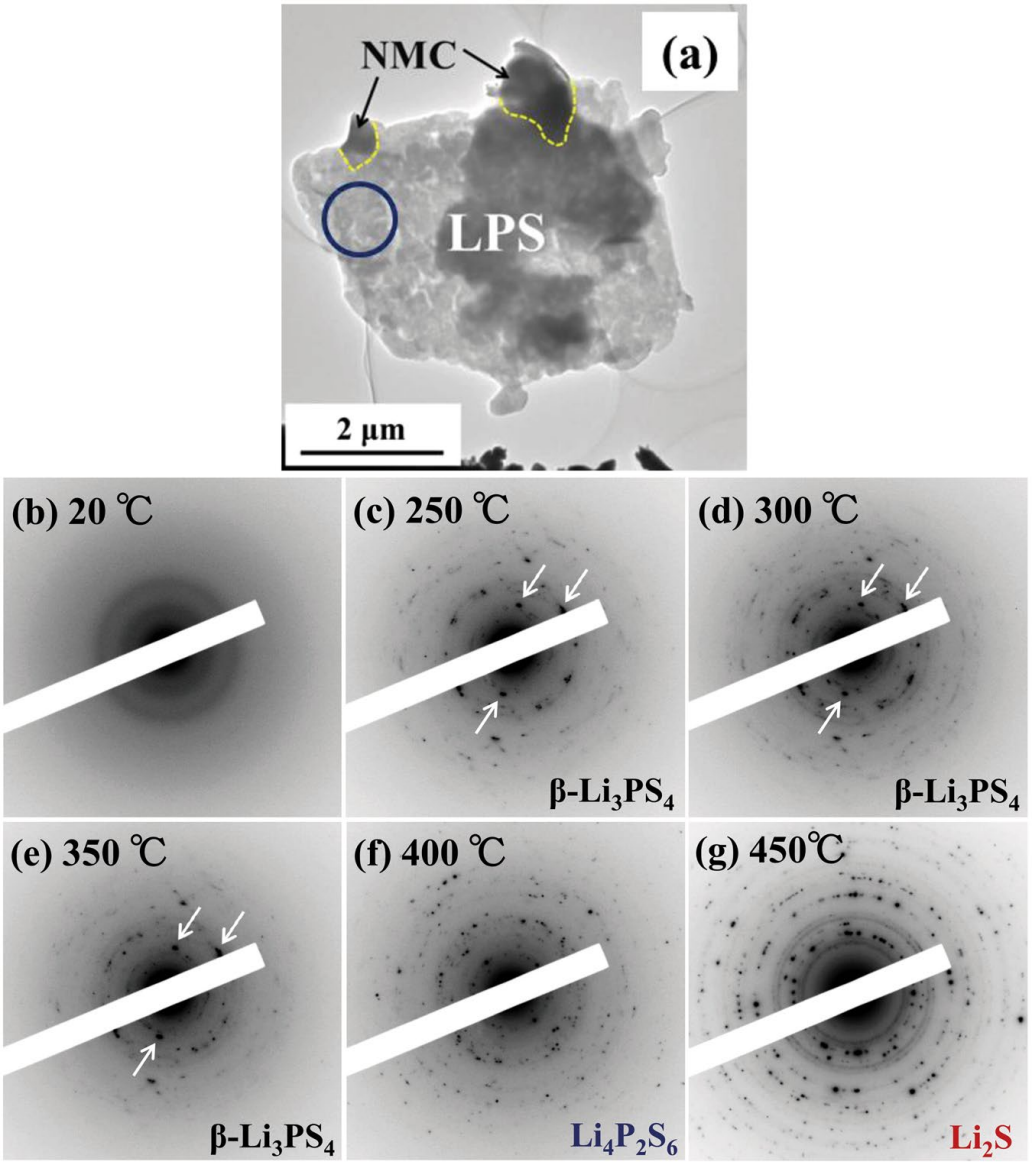
(**a**) The BF image indicating the morphology of the NMC–LPS composites after the initial charge. (**b–g**) Series of ED patterns obtained from the LPS glass area indicated by the blue circle in (**a**) upon heating from 20 °C to 450 °C.

**Figure 4:**
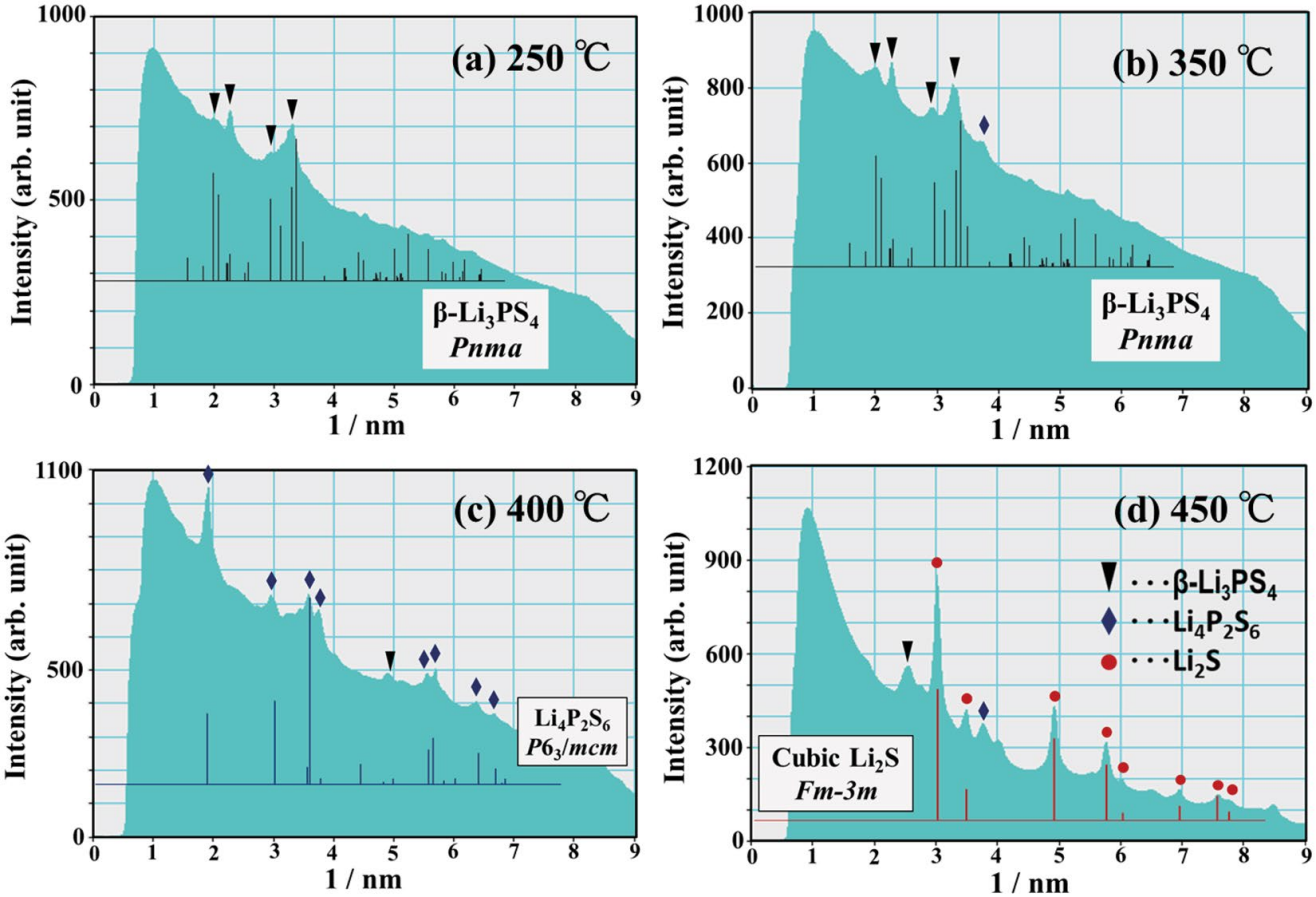
The intensity profiles of the ED patterns at (**a**) 250 °C, (**b**) 350 °C, (**c**) 400 °C, and (**d**) 450 °C, which were taken from the LPS region that had interfacial contact with NMC. Inverted triangles, diamonds, and circles indicate the precipitation of $\beta$-Li_3_PS_4_, Li_4_P_2_S_6_, and Li_2_S crystalline phases, respectively.

**Figure 5:**
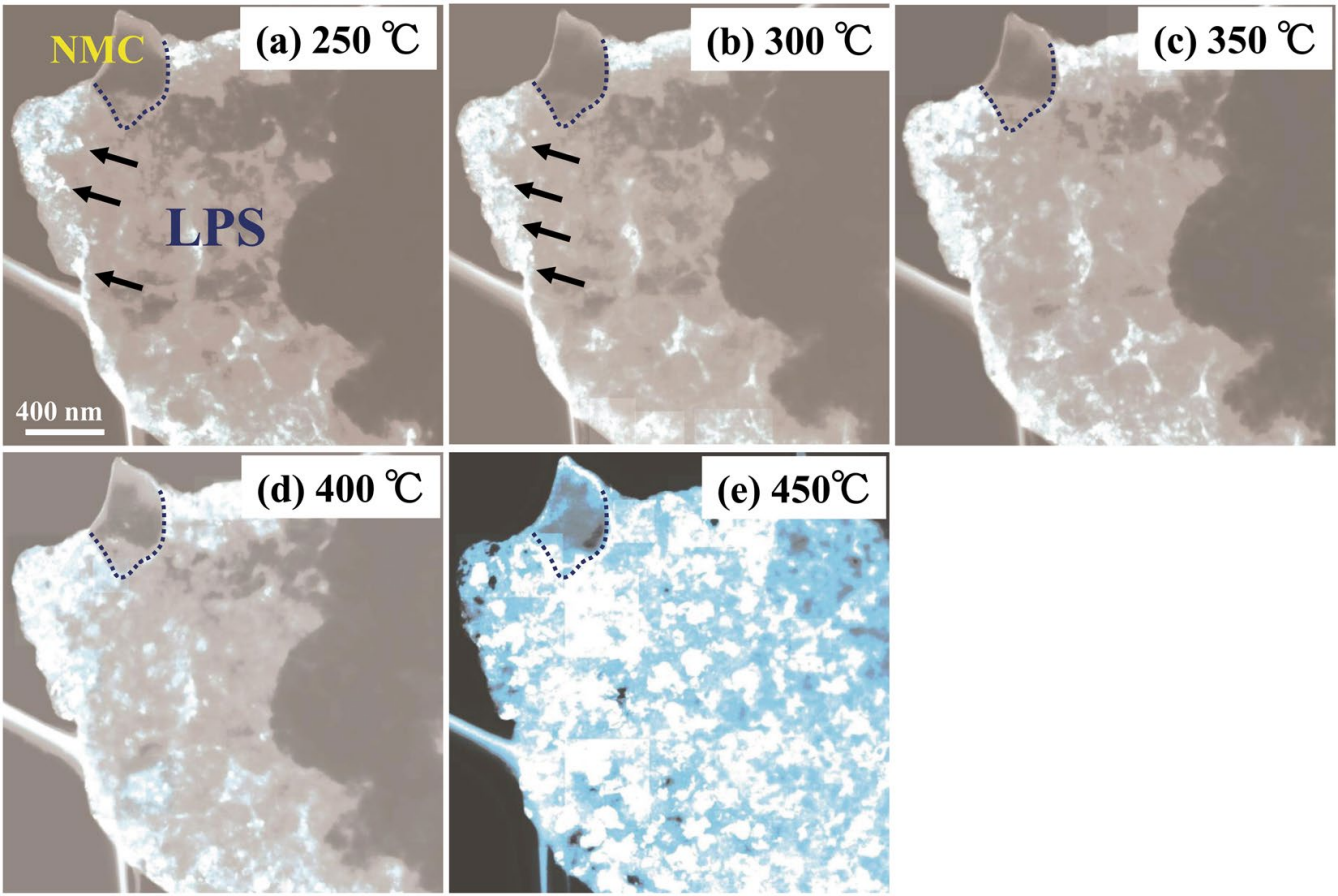
A series of superposed DF images obtained from the same LPS glass region indicated by the blue circle in Fig. 3(a).

DSC measurements were performed for the NMC–LPS composites after the charge–discharge cycles. Figure 2 shows the DSC curve for the NMC–LPS composites after the initial charge. Two conspicuous exothermic peaks were detected in the temperature range from 300 °C to 400 °C, as indicated by arrows. To clarify the origin of these exothermic peaks, *in situ* TEM observations during heating were conducted. Figure 3 shows a BF image of the LPS–NMC composite after the initial charge and a series of ED patterns obtained from the LPS region indicated by a circle in (a). In the BF image (a), the region surrounded by the yellow dotted line is the positive active material NMC, and the other region is the LPS glass electrolyte. The ED patterns in Fig. 3(b–g) were taken at 20 °C, 250 °C, 300 °C, 350 °C, 400 °C, and 450 °C, respectively. In the initial state at 20 °C, a halo pattern indicating an amorphous state was observed in the ED pattern (b). When the temperature was increased above 20 °C, crystallization started at approximately 250 °C and then gradually proceeded up to 350 °C, as shown in Fig. 3(c–e). In the temperature range from 250 °C to 350 °C, diffraction spots and Debye–Scherrer rings were observed on the relatively low angle side, as indicated by the white arrows. However, above 400 °C, crystallization significantly progressed and diffraction spots and Debye–Scherrer rings exhibiting a high intensity appeared on the high angle side. To identify the precipitated crystalline phase, intensity profiles for the ED patterns at each temperature were constructed. Figure 4 shows the intensity profiles for the ED patterns at (a) 250 °C, (b) 350 °C, (c) 400 °C, and (d) 450 °C. Below 400 °C, intensity peaks observed on the low angle side predominantly corresponded to the X-ray diffraction (XRD) profile of orthorhombic $\beta$-Li_3_PS_4_ with the space group *P*nma, as shown in Fig. 4(a,b). Thus, the crystalline phase of the material heated from 250 °C to 350 °C was identified as $\beta$-Li_3_PS_4_. When the temperature exceeded 400 °C, a significant change occurred in the intensity profiles. At 400 °C, each intensity peak was almost identical to the XRD profile of Li_4_P_2_S_6_ with the space group *P*6_3_/*mcm*. At 450 °C, although some $\beta$-Li_3_PS_4_ and Li_4_P_2_S_6_ remained, the main crystalline phase was identified as Li_2_S with the space group *F*m$\mathop{3}\limits^{\_}$m. Figure 5 shows the variations of the corresponding DF images as a function of temperature. The images in Fig. 5(a–e) were taken at 250 °C, 300 °C, 350 °C, 400 °C, and 450 °C, respectively. In DF images, bright contrasts indicate the crystallized regions. In the temperature range between 250 °C and 400 °C, nanocrystallites with a size of approximately 20–50 nm precipitated mainly on the edge of the sample, as indicated by the arrows. Conversely, above 400 °C, approximately 100-nm large nanocrystallites precipitated on both the edges and inside the sample. These nanocrystallites apparently should be Li_2_S. Therefore, crystallization in the LPS glass that has interfacial contact with NMC significantly progressed at temperature above 400 °C, together with the appearance of Li_4_P_2_S_6_ and Li_2_S.

**Figure 6:**
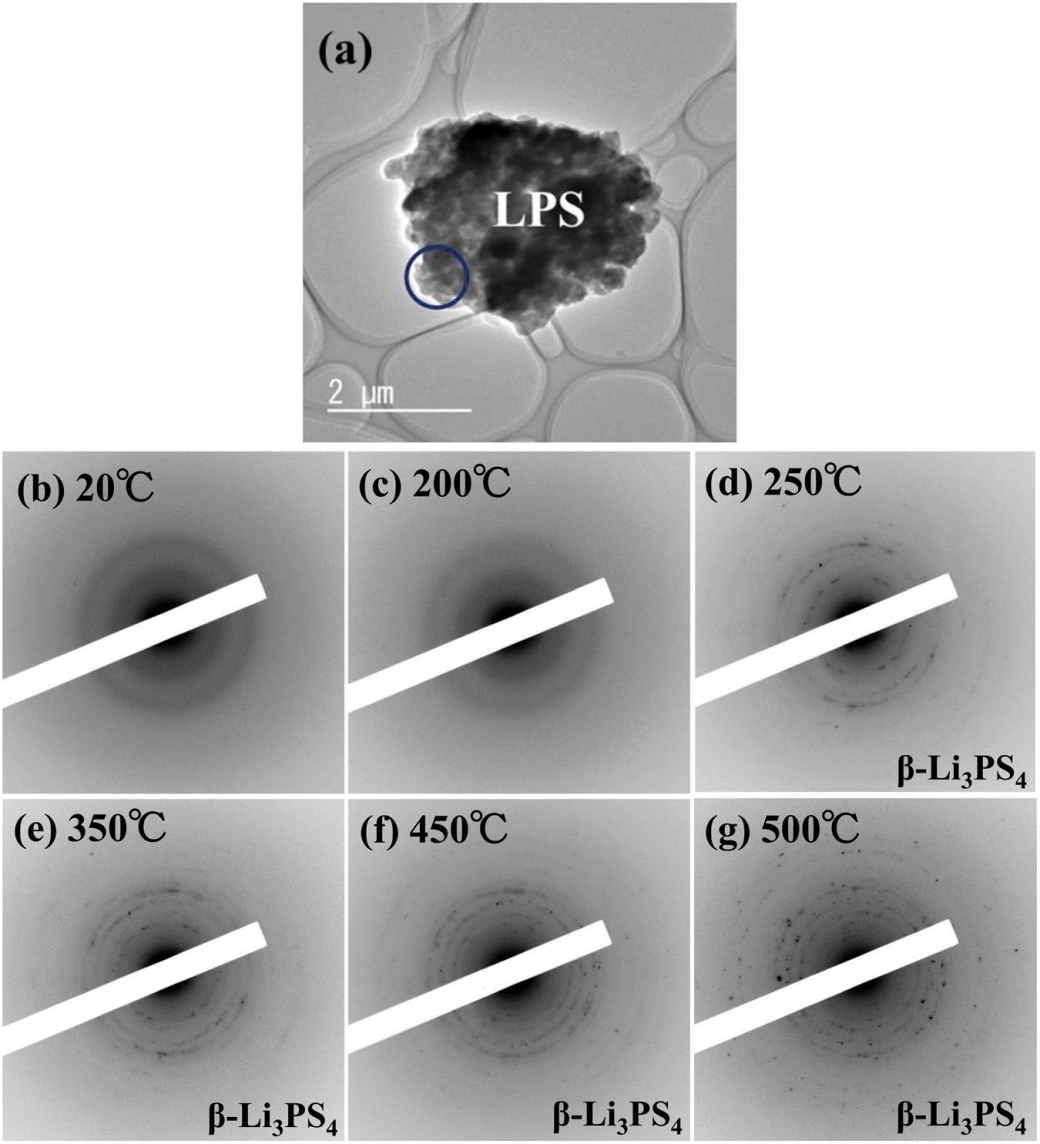
(**a**) The BF image indicating the morphology of the LPS glass without interfacial contact with NMC. (**b–g**) A series of ED patterns obtained from the area indicated by the blue circle in (**a**) upon heating from 20 °C to 500 °C.

**Figure 7:**
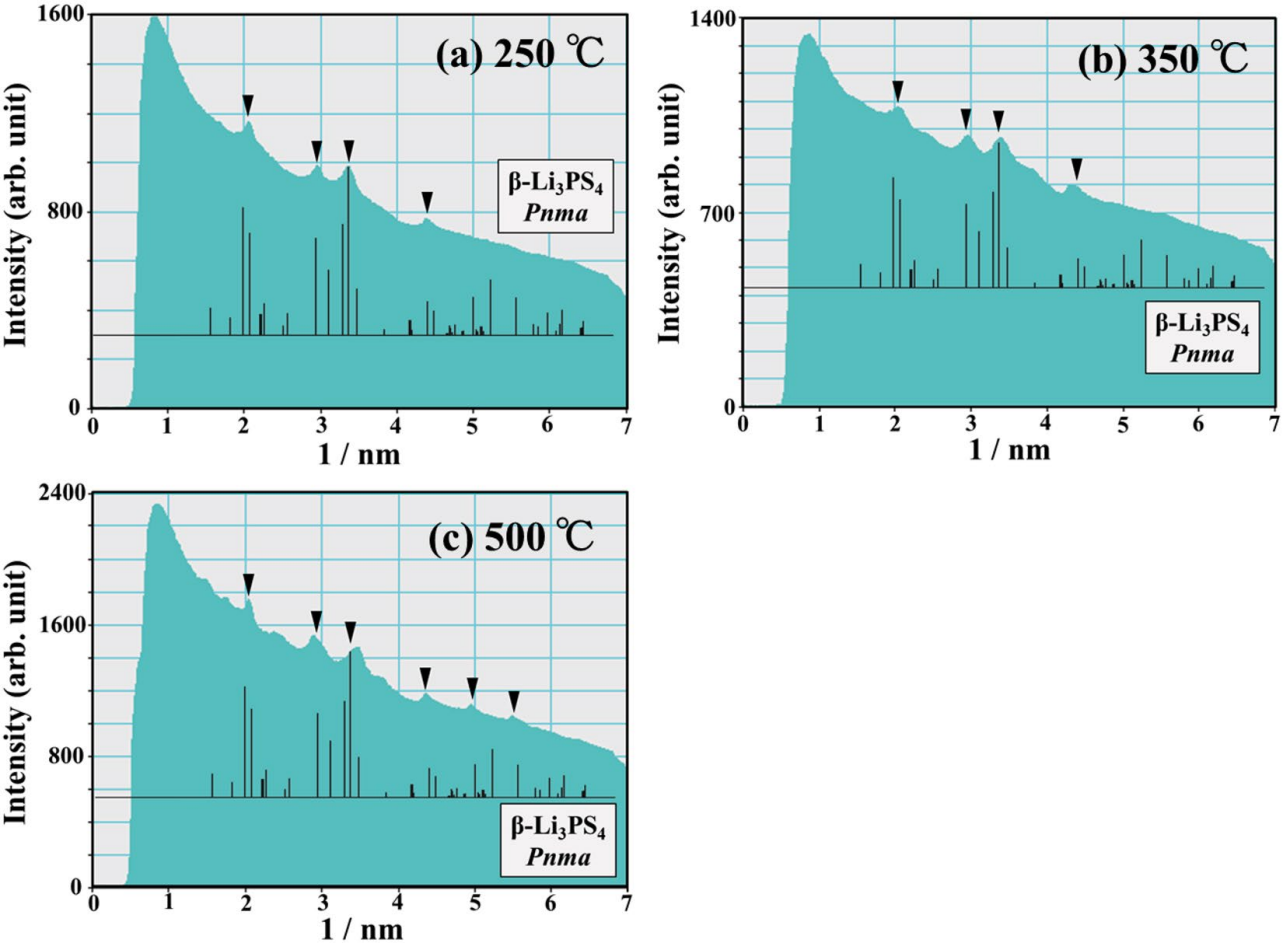
The intensity profiles of the ED patterns at (**a**) 250 °C, (**b**) 350 °C, and (**c**) 500 °C, which were taken from the LPS region without interfacial contact with NMC.

Figure 6 shows a BF image of the LPS glass without interfacial contact with NMC and a series of ED patterns obtained from the region indicated by the circle in (a). The ED patterns in Fig. 6(b–g) were taken at 20 °C, 200 °C, 250 °C, 350 °C, 450 °C, and 500 °C, respectively. In the initial state at 20 °C, the ED pattern (a) exhibited a halo pattern, which indicated an amorphous state. When the temperature was raised above 20 °C, crystallization started at approximately 250 °C and then gradually proceeded up to 500 °C, as shown in Fig. 6(c–g). Figure 7 shows the corresponding intensity profiles of the ED patterns at (a) 250 °C, (b) 350 °C, and (c) 500 °C. All the intensity peaks at each temperature were predominantly owing to $\beta$-Li_3_PS_4_. In the LPS region with no interfacial contact with NMC, only the $\beta$-Li_3_PS_4_ crystalline phase appeared. This crystallization process was different to that in the LPS region having an interfacial contact with NMC, as shown in Figs 3 and 4.

The present results showed that the LPS crystallization process differed depending on the presence of an interfacial contact with NMC. In the LPS regions adjacent to NMC, Li_4_P_2_S_6_ and Li_2_S crystalline phases precipitated at a high temperature. Reproducibility measurements of the LPS crystallization behavior in the NMC–LPS composite showed that the temperature range for the precipitation of Li_4_P_2_S_6_ and Li_2_S approximately ranged from 300 °C to 400 °C and from 370 °C to 450 °C, respectively. Therefore, it is likely that the precipitation of Li_4_P_2_S_6_ and Li_2_S were involved in two of the exothermic reaction observed in the DSC curve. On the basis of the thermodynamic parameters of the chemical reaction process, the LPS crystallization behavior in the NMC–LPS composites was investigated. For the Li_4_P_2_S_6_ crystalline phase, we examined the possibility of chemical interactions between $\beta$-Li_3_PS_4_ and NMC delithiated by charging. In the reaction process in which Li is extracted from Li_3_PS_4_ by delithiated NMC and Li_3_PS_4_ decomposes, the total energy of each substance is given by

1 $${{\rm{Li}}}_{3}{{\rm{PS}}}_{4}-{\rm{Li}}({\rm{in}}\,{\rm{NMC}})\to (1/2)\,{{\rm{Li}}}_{4}{{\rm{P}}}_{2}{{\rm{S}}}_{6}+{\rm{S}}+1.8\,{\rm{eV}}(/{\rm{f}}{\rm{.u}}.).$$

When the exothermic reaction (1) occurs and Li_4_P_2_S_6_ crystallizes, its crystallization energy could appear as heat generation. For simplicity, the electromotive force of NMC was assumed to be 4 V. Total calorific value depends on the amount of Li deficiency in NMC. Furthermore, for the Li_2_S crystalline phase that precipitated above 400 °C, chemical reactions associated with the decomposition of Li_3_PS_4_ were examined. In the reaction process in which Li_3_PS_4_ is decomposed into Li_4_P_2_S_6_ and Li_2_S, the total energy of each substance is given by

2 $$2\,{{\rm{L}}{\rm{i}}}_{3}{{\rm{P}}{\rm{S}}}_{4}\to {{\rm{L}}{\rm{i}}}_{4}{{\rm{P}}}_{2}{{\rm{S}}}_{6}+{{\rm{L}}{\rm{i}}}_{2}{\rm{S}}+{\rm{S}}-0.2\,\text{eV}(/{\rm{f.}}{\rm{u.}}).$$

When the chemical reaction (2) occurs and Li_4_P_2_S_6_ and Li_2_S crystallize, the crystallization energy of both Li_4_P_2_S_6_ and Li_2_S could appear as heat generation. Because of the relatively low heating value, the endothermic reaction (2) easily occurs. The produced sulfur in (1) and (2) may exist as a simple substance or lithium polysulfide, Li_*x*_S. Further investigation, including calculation of the crystallization energy and temperature, is currently underway for reactions (1) and (2). In addition to an interfacial contact with NMC, the difference in the LPS crystallization process shown in Figs 3–7 could be also attributed to the local bonding state of clusters and atoms in the amorphous state before crystallization. Therefore, as a next step, we will investigate the LPS crystallization behavior in the NMC–LPS composite in terms of the amorphous state in the sulfide glasses by a nano-beam electron diffraction method and differential pair distribution function analysis of high-energy XRD.

## Conclusion

To clarify the origin of the exothermic reaction in the NMC–LPS composite after initial charging, the precipitated crystalline phase in the LPS glass was investigated via *in situ* TEM observations. Notably, the LPS crystallization behavior differed depending on the presence of an interfacial contact between LPS and NMC. In the LPS glass adjacent to NMC, a $\beta$-Li_3_PS_4_ crystalline phase precipitated at approximately 250 °C, followed by the precipitation of Li_4_P_2_S_6_ and Li_2_S crystalline phases above 400 °C. Conversely, in LPS glass having no interfacial contact with NMC, only $\beta$-Li_3_PS_4_ precipitated in the temperature range from 250 °C to 500 °C. The calorific values obtained by first-principles calculations indicated that $\beta$-Li_3_PS_4_ would decompose into Li_4_P_2_S_6_ and Li_2_S in the presence of delithiated NMC during the charging process. Therefore, the interfacial contact between LPS and NMC has a significant influence on the LPS crystallization behavior and its associated exothermic reaction in the NMC–LPS composite.

## Method

### Preparation of all-solid-state cells

A positive electrode layer is composed of LiNbO_3_-coated LiNi_1/3_Mn_1/3_Co_1/3_O_2_ (Toda Kogyo Co., Ltd) powders and 75Li_2_S·25P_2_S_5_ glass electrolytes (mol %). In this study, LiNi_1/3_Mn_1/3_Co_1/3_O_2_ and the 75Li_2_S·25P_2_S_5_ glass are denoted by NMC and LPS, respectively. The LPS glass solid electrolyte was prepared from crystalline powders of Li_2_S (Mitsuwa Chemicals Co., Ltd, 99.9%) and P_2_S_5_ (Aldrich, 99%) through mechanical milling technique. Mechanical ball milling was performed to the batches (1 g) of these starting materials with a stoichiometric ratio in a zirconia pot (45 mL volume) with 500 zirconia balls (diameter in 4 mm) using a planetary ball mill apparatus (Pulverisette 7; Fritsch) at the rotation speed of 510 rpm for 45 hours^8,9^. The total conductivity of the synthesized 75Li_2_S·25P_2_S_5_ glasses is 5.0 × 10^−4^ S/cm at 25 °C. Indium foil (99.999%; Furuuchi Chemical Corp.) was used as a negative electrode. Composite positive electrode was prepared by mixing NMC and LPS particles with a weight ratio of 75:25. All-solid-state cells In/LPS/NMC were constructed in the following procedure. A bilayer pellet (diameter in 10 mm) was obtained by pressing a solid electrolyte layer and composite positive electrode layer at 360 MPa at room temperature. Indium foil was then attached to the bilayer pellet at 240 MPa. The cell was sandwiched with two stainless steel rods using as current collectors. All the processes were conducted in a glove box filled with dry argon gas (less than 1 ppm water).

### Electrochemical measurements

The all-solid-state cell In/LPS/NMC (NMC:SE = 75:25 in wt% ratio) was charged and discharged at 25 °C under a current density of 0.13 mA cm^−2^ with cutoff voltages of 1.9–3.8 V (vs. Li–In) using a charge–discharge measuring device (BTS-2004; Nagano Co Ltd., Japan). AC impedance measurements were performed for pristine and cycled cells by an impedance analyzer (SI 1260 and SI 1287; Solartoron) in a frequency range from 0.01 to 1 MHz.

### Thermal stability tests

The thermal behavior of the NMC–LPS composite was examined by differential scanning calorimeter (DSC). After charging, the cells were disassembled and the NMC–LPS composite for the DSC measurements was sealed in a stainless-steel pan in a glove box filled with dry argon gas. The DSC measurements were performed in the temperature range between 20 °C and 500 °C using a thermal analyzer (Thermo-plus 8110, Rigaku). The heating rate was 10 °C min^−1^.

### TEM observations

*In situ* TEM observations were conducted in the temperature range from 20 °C to 500 °C using a JEM-2100F field-emission TEM system with an accelerating voltage of 200 kV. To prevent the exposure of the samples to air, a double-tilt vacuum transfer TEM holder (Gatan model 648) was used. During the heating experiments, the temperature was controlled by a heater control unit (EM-SHU2, JEOL Co. Ltd). Because the crystallization process of the sulfide glass was very slow, the sample was heated in a TEM at a slow rate of 1 K/min. In addition, after the desired temperature was reached, the temperature was maintained for at least 40 min. The pressure in the TEM was approximately 1.0 × 10^−5^ Pa. The structural changes during heating of the NMC–LPS composite were examined using bright field (BF) images, dark field (DF) images, and the corresponding ED patterns. In particular, the LPS crystallization behavior was directly observed by taking DF images and superposing them (See the details in Supplementary Fig. S1). Crystalline phases in the LPS glass was identified via a computer program called “ProcessDiffraction”, in which the ED patterns can be transformed into one dimensional intensity profile^14–17^. The sample preparation procedure for TEM observations is described in our previous paper^13^.

### First-principles calculation for formation enthalpy

To evaluate the formation energies, the total energy of each element and compound were calculated based on density functional theory using the Vienna *Ab initio* Simulation Package (VASP), wherein the projector-augmented wave (PAW) potentials were taken and exchange-correlation was treated in a framework of a Perdew–Burke–Ernzerhof gradient-corrected functional^18,19^. The inner coordinates of Li, Li_4_P_2_S_6_, Li_2_S and S were optimized in their experimental lattice parameters and the cell parameters of Li_3_PS_4_ were optimized^20–23^. The wave functions were expanded into plane waves using a 400-eV cutoff, and the Brillouin zone integrations were sampled by 8 × 8 × 8, 3 × 5 × 5, 5 × 5 × 5, 3 × 3 × 1 and 3 × 3 × 5 Monkhorst–Pack k-point grids.

## Electronic supplementary material

Supplementary Information

## References

[1] Kato Y (2016). High-power all-solid-state batteries using sulfide superionic conductors. Nat. Energy.

[2] Kamaya N (2011). A lithium superionic conductor. Nat. Mater..

[3] Kuhn A, Duppel V, Lotsch BV (2013). Tetragonal Li_10_GeP_2_S_12_ and Li_7_GePS_8_ - exploring the Li ion dynamics in LGPS Li electrolytes. Energy Environ. Sci..

[4] Bron P (2013). Li_10_SnP_2_S_12_: An Affordable Lithium Superionic Conductor. J. Am. Chem. Soc..

[5] Yamane H (2007). Crystal structure of a superionic conductor, Li_7_P_3_S_11_. Solid State Ion..

[6] Seino Y, Ota T, Takada K, Hayashi A, Tatsumisago M (2014). A sulphide lithium super ion conductor is superior to liquid ion conductors for use in rechargeable batteries. Energy Environ. Sci..

[7] Ujiie S, Hayashi A, Tatsumisago M (2013). Mater. Renew. Sustain. Energy.

[8] Mizuno F, Hayashi A, Tadanaga K, Tatsumisago M (2005). New, Highly Ion-Conductive Crystals Precipitated from Li_2_S–P_2_S_5_ Glasses. Adv. Mater..

[9] Hayashi A, Minami K, Ujiie S, Tatsumisago M (2010). Preparation and ionic conductivity of Li_7_P_3_S_11−z_ glass-ceramic electrolytes. J. Non-Cryst. Solids.

[10] Mizuno F, Hayashi A, Tadanaga K, Tatsumisago M (2006). High lithium ion conducting glass-ceramics in the system Li_2_S–P_2_S_5_. Solid State Ion..

[11] Hayashi A, Hama S, Minami T, Tatsumisago M (2003). Formation of superionic crystals from mechanically milled Li_2_S–P_2_S_5_ glasses. Electrochem. Commun..

[12] Tsukasaki H, Mori S, Morimoto H, Hayashi A, Tatsumisago T (2017). Direct observation of a non-crystalline state of Li_2_S–P_2_S_5_ solid electrolytes. Sci. Rep..

[13] Tsukasaki H (2017). Analysis of structural and thermal stability in the positive electrode for sulfide-based all-solid-state lithium batteries. J. Power Sources.

[14] Lábár JL (2005). Consistent indexing of a (set of) single crystal SAED pattern(s) with the ProcessDiffraction program. Ultramicroscopy.

[15] Lábár JL (2008). Electron diffraction based analysis of phase fractions and texture in nanocrystalline thin films, part I: Principles, *Microsc*. Microanal..

[16] Lábár JL (2009). Electron diffraction based analysis of phase fractions and texture in nanocrystalline thin films, Part II: Implementation. Microsc. Microanal..

[17] Lábár JL (2012). Electron diffraction based analysis of phase fractions and texture in nanocrystalline thin films, part III: Application examples, *Microsc*. Microanal..

[18] Kresse G (1993). & Hafner, *Ab initio* molecular dynamics for liquid metals. Phys. Rev. B.

[19] Perdew JP, Burke K, Ernzerhof M (1996). Generalized Gradient Approximation Made Simple. Phys. Rev. Lett..

[20] Smith HG, Berliner R, Jorgensen JD, Nielsen M, Trivisonno J (1990). Pressure effects on the martensitic transformation in metallic lithium. Phys. Rev. B.

[21] Mercier R, Malugani JP, Fahys B, Douglande J, Robert G (1982). Synthese, structure cristalline et analyse vibrationnelle de l’hexathiohypodiphosphate de lithium Li_4_P_2_S_6_. J. Solid State Chem..

[22] Kubel F, Bertheville B, Bill H (1999). Crystal structure of dilithiumsulfide, Li_2_S. Z. Kristallogr..

[23] Abrahams SC (1955). The crystal and molecular structure of orthorhombic sulfur. Acta Crystallogr..

