# Supplementary material for: Crystallization behavior of the Li2S–P2S5 glass electrolyte in the LiNi1/3Mn1/3Co1/3O2 positive electrode layer
Source: Sci Rep. 2018 Apr 18;8:6214. doi: 10.1038/s41598-018-24524-7 (PMC5906461; doi:10.1038/s41598-018-24524-7)
Supplement: Supplementary file 1 — Supplementary Information [file 41598_2018_24524_MOESM1_ESM.docx]

SUPPLEMENTARY INFORMATION TO

**Crystallization behavior of the Li_2_S–P_2_S_5_ glass electrolyte in the LiNi_1/3_Mn_1/3_Co_1/3_O_2_ positive electrode layer**

Hirofumi Tsukasaki^*^, Yota Mori, Misae Otoyama, So Yubuchi, Takamasa Asano, Yoshinori Tanaka, Takahisa Ohno, Shigeo Mori, Akitoshi Hayashi, Masahiro Tatsumisago

(*Corresponding Author)


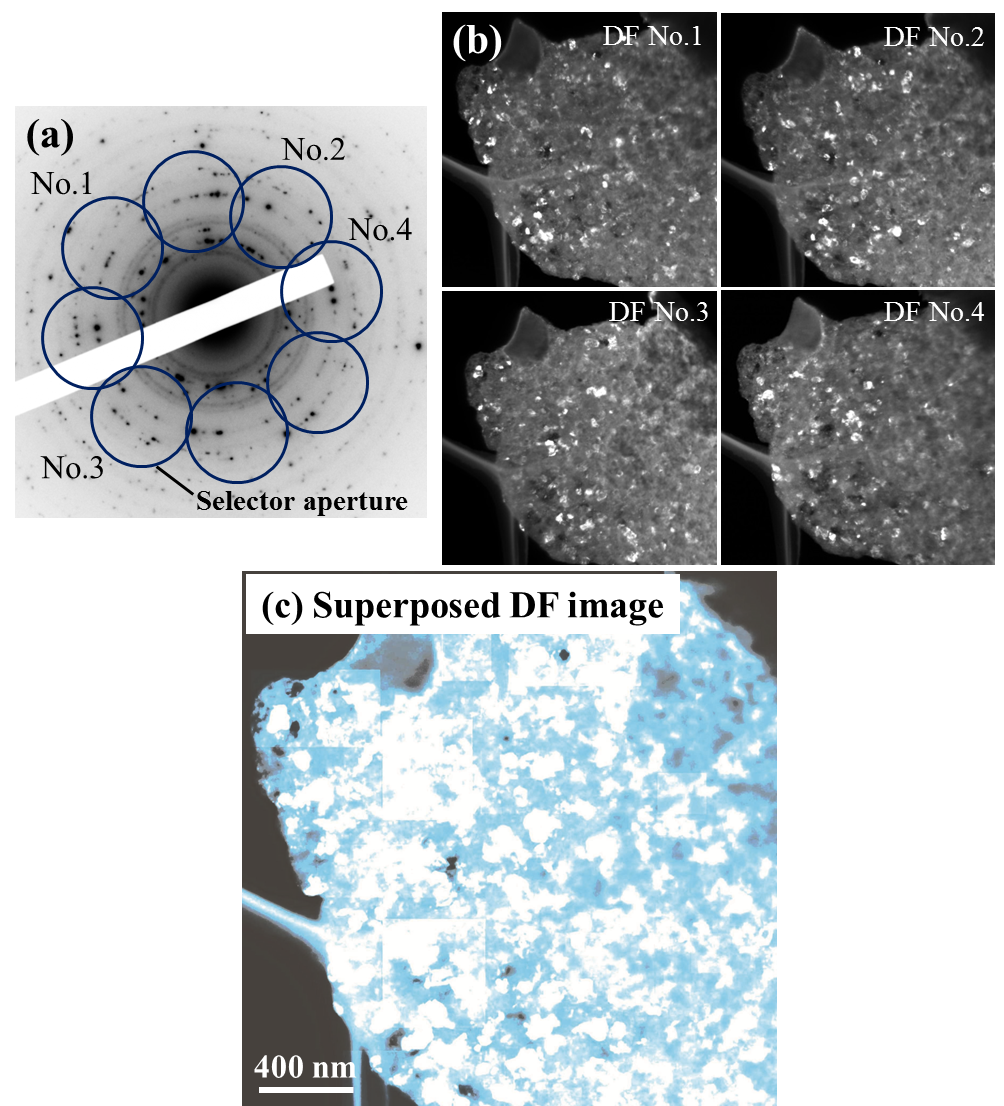


**Figure S1** shows the procedure for observing the crystallization behavior in LPS glass. First, an ED pattern from the crystallized LPS region was taken, which shows Debye rings (Fig. S1(a)). The blue circles indicate the positions at which a selector aperture is inserted. Subsequently, a DF image is taken by selecting some of the diffraction spots in the ED pattern. Finally, several DF images are taken of all the diffraction spots that form the Debye ring, as shown in Fig. S1(b). DF images No.1–4 were taken using the respective spots No.1–4, as indicated by blue circles in (a). The bright-contrast regions in these DF images are different. Then, by superposing all the DF images, the regions corresponding to the diffraction spots can be visualized as bright-contrast regions. The spatial distribution and sizes of precipitated nanocrystallites can be clearly observed, as shown in Fig. S1(c).
